# Supplementary material for: Insecticidal characteristics and structural identification of the potential active compounds from Streptomyces sp. KR0006: Strain improvement through mutagenesis
Source: PLoS One. 2022 Sep 26;17(9):e0274766. doi: 10.1371/journal.pone.0274766 (PMC9512179; doi:10.1371/journal.pone.0274766)
Supplement: S1 Table — To estimate the larvicidal activity, Chinese cabbage leaf disks (diameter 3 cm) were dipped for 30 seconds in the recommended concentration of insecticide solutions. Once the leaf disks were surface-dried they were inoculated with P. xylostella 2nd instars (10 larvae/disk). Distilled water was used as untreated control. Letters in each column indicate significant differences in the Fisher’s LSD test (P < 0.05). (PDF) [file pone.0274766.s001.pdf]

Replications	Larval mortality (%)		
	Untreated control	Biological control agent (*Bacillus thuringiensis* 4g L^{-1})	Chemical insecticide (Diflubenzuron 0.25g L^{-1})
1	0±0a	100±0a	100±0a
2	0±0a	100±0a	100±0a
3	0±0c	100±0a	100±0a
