# Supplementary material for: Insecticidal characteristics and structural identification of the potential active compounds from Streptomyces sp. KR0006: Strain improvement through mutagenesis
Source: PLoS One. 2022 Sep 26;17(9):e0274766. doi: 10.1371/journal.pone.0274766 (PMC9512179; doi:10.1371/journal.pone.0274766)
Supplement: Attachment — Submitted filename: PONE-D-22-11701.pdf [file pone.0274766.s002.pdf]

PLOS ONE

Insecticidal characteristics and s tructural identification of the potential active compounds from Streptomyces sp. KR0006: Strain improvement through mutagenesis.

--Manuscript Draft--

**Manuscript Number:**	PONE-D-22-11701
**Article Type:**	Research Article
**Full Title:**	Insecticidal characteristics and s tructural identification of the potential active compounds from Streptomyces sp. KR0006: Strain improvement through mutagenesis.
**Short Title:**	Insecticidal activity of the secondary metabolites from actinomycetes.
**Corresponding Author:**	Mirjalol Umurzokov<br>Chungnam National University College of Agriculture and Life Sciences<br>Daejeon, Korea KOREA, REPUBLIC OF
**Keywords:**	Actinomycetes, Insecticidal activity, Plutella xylostella, Streptomyces sp. KR0006, UV-Mutation
**Abstract:**	Pest control by biological means is an effective, eco-friendly, and promising method that typically involves naturally derived compounds from actinomycetes. Thus, the present study aimed to screen, characterize, and identify the structure of insecticidal compounds from Streptomyces sp. KR0006 and increase the activity through mutagenesis. In the examination of the insecticidal activity level of the isolates, Streptomyces sp. KR0006 metabolite showed significant activity against larvae and moths of Plutella xylostella . Taxonomic analyses of the 16S rRNA gene sequences revealed that the isolated KR0006 strain tended to be 99% consistent with Streptomyces cinereoruber strain NBRC 12756. Three active compounds isolated from the culture filtrate KR0006 were purified by solvent partition, mid-pressure liquid chromatography (MPLC), Sephadex LH20 column chromatography, and high-performance liquid chromatography (HPLC). By performing 1 H-NMR, 13 C-NMR, and 2D-NMR experiments, and high-resolution electrospray ionization mass spectrometry analysis, the 316-HP2, 316-HP3, and 316-HP5 compounds were inferred as antimycin A3a (MW, 519.; C 26 H 36 N 2 O 9), antimycin A8a (MW, 534; C 27 H 38 N 2 O 9), and antimycin A1a (MW, 548; C 28 H 40 N 2 O 9) respectively. Mutant U67 obtained from exposure to ultraviolet (UV) irradiation (254 nm, height 17 cm) for 70 seconds resulted in a 70% more larval mortality than that of the initial wild culture. The second mutation of the culture broth enhanced insecticidal activity by 80 and 100 % compared with the first mutation and initial medium, respectively. Our study found that Streptomyces sp. KR0006 strain produces insecticidal active compounds and could be put a practical use in pest management.
**Order of Authors:**	Young Sook Kim
	Mirjalol Umurzokov
	Kwang Min Cho
	Jung Sup Choi
	Kee Woong Park
**Additional Information:**	
**Question**	**Response**
**Financial Disclosure**<br><br>Enter a financial disclosure statement that describes the sources of funding for the work included in this submission. Review the submission guidelines for detailed requirements. View published research articles from *PLOS ONE* for specific	This work was supported by Korea Institute of Planning and Evaluation for Technology in Food, Agriculture and Forestry (IPET) through Agricultural Machinery/Equipment Localization Technology Development Program, funded by Korea Institute of Planning and Evaluation for Technology in Food, Agriculture and Forestry (IPET) (321056-05).

Powered by Editorial Manager® and ProduXion Manager® from Aries Systems Corporation

examples.<br><br>This statement is required for submission and **will appear in the published article** if the submission is accepted. Please make sure it is accurate.<br><br>**Unfunded studies**<br>Enter: *The author(s) received no specific funding for this work.*<br><br>**Funded studies**<br>Enter a statement with the following details:<br>• Initials of the authors who received each award<br>• Grant numbers awarded to each author<br>• The full name of each funder<br>• URL of each funder website<br>• Did the sponsors or funders play any role in the study design, data collection and analysis, decision to publish, or preparation of the manuscript?<br>• **NO** - Include this sentence at the end of your statement: *The funders had no role in study design, data collection and analysis, decision to publish, or preparation of the manuscript.*<br>• **YES** - Specify the role(s) played.<br><br>* typeset	
**Competing Interests**<br><br>Use the instructions below to enter a competing interest statement for this submission. On behalf of all authors, disclose any competing interests that could be perceived to bias this work—acknowledging all financial support and any other relevant financial or non-financial competing interests.<br><br>This statement is required for submission and **will appear in the published article** if the submission is accepted. Please make sure it is accurate and that any funding sources listed in your Funding Information later in the submission form are also declared in your Financial Disclosure statement.	The authors have declared that no competing interests exist.

View published research articles from [*PLOS ONE*] for specific examples.<br><br>**NO authors have competing interests**<br><br>Enter: *The authors have declared that no competing interests exist.*<br><br>**Authors with competing interests**<br><br>Enter competing interest details beginning with this statement:<br><br>*I have read the journal's policy and the authors of this manuscript have the following competing interests: [insert competing interests here]*<br><br>* typeset	
**Ethics Statement**<br><br>Enter an ethics statement for this submission. This statement is required if the study involved:<br><br>• Human participants<br>• Human specimens or tissue<br>• Vertebrate animals or cephalopods<br>• Vertebrate embryos or tissues<br>• Field research<br><br>Write "N/A" if the submission does not require an ethics statement.<br><br>General guidance is provided below. Consult the submission guidelines for detailed instructions. **Make sure that all information entered here is included in the Methods section of the manuscript.**	The diamondback moths (Plutella xylostella) and larvae were reared on cabbage leaves and maintained at 25℃and 65% relative humidity with a 12 h light/12 h dark cycle.

**Format for specific study types**<br><br>**Human Subject Research (involving human participants and/or tissue)**<br>• Give the name of the institutional review board or ethics committee that approved the study<br>• Include the approval number and/or a statement indicating approval of this research<br>• Indicate the form of consent obtained (written/oral) or the reason that consent was not obtained (e.g. the data were analyzed anonymously)<br><br>**Animal Research (involving vertebrate animals, embryos or tissues)**<br>• Provide the name of the Institutional Animal Care and Use Committee (IACUC) or other relevant ethics board that reviewed the study protocol, and indicate whether they approved this research or granted a formal waiver of ethical approval<br>• Include an approval number if one was obtained<br>• If the study involved *non-human primates*, add *additional details* about animal welfare and steps taken to ameliorate suffering<br>• If anesthesia, euthanasia, or any kind of animal sacrifice is part of the study, include briefly which substances and/or methods were applied<br><br>**Field Research**<br><br>Include the following details if this study involves the collection of plant, animal, or other materials from a natural setting:<br>• Field permit number<br>• Name of the institution or relevant body that granted permission	
**Data Availability**<br><br>Authors are required to make all data underlying the findings described fully available, without restriction, and from the time of publication. PLOS allows rare exceptions to address legal and ethical concerns. See the PLOS Data Policy and FAQ for detailed information.	Yes - all data are fully available without restriction

A Data Availability Statement describing where the data can be found is required at submission. Your answers to this question constitute the Data Availability Statement and **will be published in the article**, if accepted.<br><br>**Important:** Stating 'data available on request from the author' is not sufficient. If your data are only available upon request, select 'No' for the first question and explain your exceptional situation in the text box.<br><br>Do the authors confirm that all data underlying the findings described in their manuscript are fully available without restriction?	
**Describe where the data may be found in full sentences. If you are copying our sample text, replace any instances of XXX with the appropriate details.**<br><br>• If the data are **held or will be held in a public repository**, include URLs, accession numbers or DOIs. If this information will only be available after acceptance, indicate this by ticking the box below. For example: *All XXX files are available from the XXX database (accession number(s) XXX, XXX.).*<br>• If the data are all contained **within the manuscript and/or Supporting Information files**, enter the following: *All relevant data are within the manuscript and its Supporting Information files.*<br>• If neither of these applies but you are able to provide **details of access elsewhere**, with or without limitations, please do so. For example:<br><br>*Data cannot be shared publicly because of [XXX]. Data are available from the XXX Institutional Data Access / Ethics Committee (contact via XXX) for researchers who meet the criteria for access to confidential data.*<br><br>*The data underlying the results presented in the study are available from (include the name of the third party*	No

and contact information or URL).<br>• This text is appropriate if the data are owned by a third party and authors do not have permission to share the data.<br><br>* typeset	
Additional data availability information:	

Manuscript Click here to access/download;Manuscript;Manuscript.docx

Research article

Insecticidal characteristics and structural identification of the potential active compounds from *Streptomyces* sp. KR0006: Strain improvement through mutagenesis.

Young Sook Kim[1†], Umurzokov Mirjalol[2†], Kwang Min Cho[3] Jung Sup Choi[1*] and Kee Woong Park[2,3*]

[1]Eco-friendly and New Materials Research Center, Korea Research Institute of Chemical Technology, Daejeon 34114, Korea

[2]Department of Crop Science, College of Agriculture and Life Sciences, Chungnam National University 34134, Daejeon, Korea

[3]Daeseungbiofarm Co., Ltd., Daejeon 34127 Korea

[†]These authors contributed equally to this work.

[*]Corresponding authors:

Jung Sup Choi: E-mail) jschoi@krict.re.kr

Kee Woong Park: E-mail) parkkw@cnu.ac.kr

Abstract

Pest control by biological means is an effective, eco-friendly, and promising method that typically involves naturally derived compounds from actinomycetes. Thus, the present study aimed to screen, characterize, and identify the structure of insecticidal compounds from *Streptomyces* sp. KR0006 and increase the activity through mutagenesis. In the examination of the insecticidal activity level of the isolates, *Streptomyces* sp. KR0006 metabolite showed significant activity against larvae and moths of *Plutella xylostella*. Taxonomic analyses of the 16S rRNA gene sequences revealed that the isolated KR0006 strain tended to be 99% consistent with *Streptomyces cinereoruber* strain NBRC 12756. Three active compounds isolated from the culture filtrate KR0006 were purified by solvent partition, mid-pressure liquid chromatography (MPLC), Sephadex LH20 column chromatography, and high-performance liquid chromatography (HPLC). By performing ^{1}H-NMR, ^{13}C-NMR, and 2D-NMR experiments, and high-resolution electrospray ionization mass spectrometry analysis, the 316-HP2, 316-HP3, and 316-HP5 compounds were inferred as antimycin A3a (MW, 519.; $C_{26}H_{36}N_2O_9$), antimycin A8a (MW, 534; $C_{27}H_{38}N_2O_9$), and antimycin A1a (MW, 548; $C_{28}H_{40}N_2O_9$) respectively. Mutant U67 obtained from exposure to ultraviolet (UV) irradiation (254 nm, height 17 cm) for 70 seconds resulted in a 70% more larval mortality than that of the initial wild culture. The second mutation of the culture broth enhanced insecticidal activity by 80 and 100 % compared with the first mutation and initial medium, respectively. Our study found that *Streptomyces* sp. KR0006 strain produces insecticidal active compounds and could be put a practical use in pest management.

Keywords: Actinomycetes, Insecticidal activity, *Plutella xylostella*, *Streptomyces* sp. KR0006, UV-Mutation

1. Introduction

Chemical insecticides are most widely used to protect plants from harmful insects since they have high efficiency in pest control. At the same time, there is a common concern associated with the negative impact of insecticides on the environment, humans, and non-target organisms due to chemical pollution, accumulation of toxic residues in soil and water, and development of pesticide resistance in pests [1, 2]. In addition, the use of synthetic pesticides leads to the reproduction of pests and the proliferation of secondary pests and ultimately disrupts the ecosystem's ecological balance by killing natural pest predators [3, 4]. For these reasons, there is a need to develop and implement safe and environmentally friendly biological approaches to conventional chemical practices in pest management.

Biopesticidal metabolites as part of biological control in pest management have a great interest and are being studied among researchers as they are eco-friendly, selective, degradable, and non-toxic natural products [3, 5-7]. Among the biological control agents derived from microorganisms, actinomycetes represent one of the most important and widely studied microbial resources because they produce various forms of active compounds for use as pesticides [8-10]. *Streptomyces* spp. is a major group of actinomycetes inhabiting the soil that shows insecticidal activity. Kim, Choi (7) reported that active compounds isolated from *Streptomyces* exhibited insect growth regulatory and insecticidal activities against *Aedes albopictus* and *Plutella xylostella*. Prasinons, avermectin doramectin, milbemycin, nanchangmycin, spinosad, and dianemycin have been derived as active compounds from the genus *Streptomyces* spp., against a variety of pest insects [11-14].

From the economic point of view, the production and application of the secondary metabolites from wild-type strains do not justify themselves much. Therefore, it is necessary to find ways to improve activity and achieve several times higher efficiency of the isolated secondary metabolites. UV irradiation method is the most convenient, safe, and widely used in

microbial strain improvement [15-17].

Thus, the present study aimed to screen, characterize, and identify the structure of insecticidal compounds from *Streptomyces* KR0006 and increase the activity through mutagenesis.

2. Materials and Methods

2.1. Isolation of Actinomycetes and fermentative incubation

A total of 850 *Actinomycetes* were isolated from the soil collected in the Jeongseon area, Gangwon-do, Korea using HV (humic acid-vitamin) and Bennet's agar medium. The Actinomycetes strains were fermented in a 500ml baffled Erlenmeyer flask containing 100ml M3 culture media (6 g $CaCO_3$, 20 g glucose, 20 g soytone, 40 g soluble starch, and 1 L distilled water, pH of 6.9), which were incubated on a rotary shaker (250 rpm) at 27℃ for 7 days. Fermentative broth cultures were subjected to centrifugation at 8000 rpm for 10 minutes and the supernatant was used for further study of insecticidal activity.

2.2. Experimental Insect

The diamondback moths (*Plutella xylostella)* and larvae were reared on cabbage leaves and maintained at 25℃ and 65% relative humidity with a 12 h light/12 h dark cycle.

2.3. Bio-insecticidal assay

Insecticidal activities of actinobacterial culture media against *P. xylostella* larvae were evaluated using cabbage leaf disk dipping assay and whole plant assay. Chinese cabbage leaf disks (diameter 3 cm) were dipped for 30 seconds in an undiluted culture filtrate. Distilled water was used as an untreated control. Once the leaf disks dried, they were inoculated with *P. xylostella* 2nd instars (10 larvae/disk). The number of dead larvae was checked at 24 h intervals

and calculated 3days after treatment. In the case of the whole plant assay, 10mL culture filtrate was sprayed on the foliage of Chinese cabbage (2 plants per pot) growing for 10 days under greenhouse conditions (30/25 ± 5℃, light/ dark = 14/10 h). Then, plants were inoculated with *P. xylostella* 2nd instars (10 larvae/plant). The larvicidal and antifeeding activities were evaluated in the cabbage seedlings at 5 DAT. All experiments were independently repeated three times.

The effect of ovipositional deterrent and controlling the larvae of *P. xylostella* was investigated using Chinese cabbage (40 seedlings per tray) grown for 10 days in a greenhouse. The culture filtrate 50mLwas first sprayed on the cabbage seedlings and the tray was put in a plastic box (30x25x30cm) and then inoculated with a diamondback moth (50 adults/box). 6 days after inoculation (DAT), the culture filtrate was secondarily applied to half of the tray. The fecundity and hatching rate and larval mortality were determined by comparing the first and secondary treatments with untreated control. All experiments were independently repeated three times.

2.4. Statistical analysis

One-Way ANOVA statistical analysis was performed using OriginPro 8.1 [18]. The Fisher's LSD test was used to compare the mean value, and the means values < 0.05 were considered statistically significant.

2.5. Taxonomic characteristics of the strain

To conduct taxonomic characteristics of the strain KR0006, 16S rRNA gene nucleotide sequencing, and PCR amplification analyses were performed as reported by Bo, Kim (19) PCR products were commissioned to Macrogen Co., Ltd. (Daejeon, Korea) to perform gene

sequencing. Obtained sequences of the secured 16S rRNA gene were analyzed through the BLASTn program of NCBI (http://www.ncbi.nlm.nih.gov) and were aligned with similar sequences using the CLUSTAL W program (MEGA 6.0.). The phylogenetic tree of the strain was created using the Neighbor-joining method in the MEGA 6 program [20, 21]. The bootstrap analyses were implemented with 1000 replications to evaluate the stability of constructed phylogenetic tree.

2.6. Purification and structural identification of the active compounds

To separate the active substance produced by the strain KR0006, the culture filtrate was first extracted using ethyl acetate (EtOAc) as reported by Kim, Bo (22). EtOAc fraction was purified by Mid-pressure liquid chromatography (MPLC) system eluted with a gradient of increasing methanol concentration (70% to 100%) in water, followed by Sephadex LH-20 (Phamarcia, Uppsala, Sweden) column chromatography eluted with 100% methanol.

The insecticidal activity of each obtained fraction was evaluated by conducting the leaf disk-feeding assay (see bio-insecticidal assay). The active fraction was finally purified by reversed-phase high-performance liquid chromatography with C18 (Atlantis T3; WATERS) column using 75% acetonitrile containing 0.02% trifluoroacetic acid.

NMR spectra were recorded on a Bruker AVANCE HD 800 NMR spectrometer (800 MHz for ^{1}H and 200 MHz for ^{13}C) and Bruker AVANCE HD 700 NMR spectrometer (700 MHz for ^{1}H and 175 MHz for ^{13}C) at Korea Basic Science Institute (KBSI) in Ochang, Korea. NMR spectra were recorded in DMSO-d_6 and chemical shifts were referenced to the residual solvent signal (δC 39.5, δH 2.50). High-resolution electrospray ionization mass spectrometry (HR-ESIMS) data were acquired on a Q-TOF mass spectrometer (SYNAPT G2, Waters) at KBSI, Ochang, Korea.

2.7. Strain activity improvement through Mutagenesis–UV mutation

The strain KR0006 spores was exposed to UV rays at a distance of 17 cm from the UV lamp (DESAGA, Sarstedt-Gruppe, MinUVIS UV Lamp) (λ = 254nm) for 30, 45, 60, and 90 seconds. All these exposures were performed in a dark room to avoid any photoreaction in the production of mutants. The spores were then spread in plates containing Bennet's agar. The plates were incubated at 27℃ for 72 hours. The method employed for UV mutagenesis was described earlier by Khattab (23) and Wang, Huang (24).

The survival and lethality rates were assessed after UV exposure and survived mutant colonies were cultured on an M3 liquid medium as described earlier (see fermentative incubation). Once mutant culture supernatants were harvested, the activity of each mutant was screened on the larvae of *P. xylostella* by conducting the leaf disk-feeding assay as described earlier (see bio-insecticidal assay).

The mutant strain that exhibited the highest insecticidal activity was selected and subjected to second-time mutagenesis aimed to increase the activity of the mutant obtained from the first mutation. The procedures of mutation and screening taken in the first mutation were carried out as exactly in the second mutation.

3. Results

3.1. Insecticidal activities of Streptomyces isolate

As a result of screening 850 actinomycetes for their insecticidal activity on the larvae of *P. xylostella* using the leaf disc feeding assay, high insecticidal activity was found in 6 actinomycetes (KRA18-68, KRA18-104, KRA18-290, KR0512, KR0006, KR0603) (Fig. 1). Among the six strains, the KR0006 strain showed high insecticidal activities with mortalities of 100% after 3 days of treatment and was selected for further studies.

In an experiment using whole plants, it was confirmed that the larvae control effect was 90%

in 5-fold dilution at 5 DAT, but the mortality rate was significantly reduced when 10-fold dilution was treated. It was confirmed that the insecticidal activity of the KR0006 culture filtrate was concentration-dependent and the insecticidal effect increased with time after treatment (Fig. 2). In addition, even in cabbage seedlings treated with high-concentration KR0006 culture filtrate, some feeding marks appeared due to larvae activity at the initial stage of treatment, but the larvae were confirmed to be completely dead (Fig. 3).

The ovipositional deterrent activity experiments revealed that the occurrence of the larvae due to inhibition of fecundity and hatching of diamondback moths was considerably less than that of the untreated control. Also, the feeding marks caused by larvae after the secondary treatment of the culture filtrate were similar to those of the primary treatment, which is because the feeding ability of the larvae was inhibited by the secondary treatment of culture filtrate (Fig. 4). Thereby, we confirmed that the culture filtrate of the KR0006 strain showed insecticidal activity by inhibiting the oviposition of moths and the growth and development of larvae.

3.2. Taxonomic classification of the strain KR0006

The PCR technique using universal primers was performed to obtain the nucleotide sequence of the 16S rRNA gene of the KR0006 strain. An obtained nucleotide sequence was compared with the nucleotide sequence of other *Streptomyces* strains through the BLASTn program of NCBI (http://www.ncbi.nlm.nih.gov). Results of comparative analysis with similar bacterial strains through 16S rRNA sequencing analysis showed that this strain tended to be 99% consistent with the *Streptomyces cinereoruber* NBRC 12756 strain (Fig. 5).

3.3. Purification and structural determination of the active compounds

The fermentation broth (10 liters) of strain KR0006 was partitioned between the EtOAc and H_2O. The EtOAc soluble portion was further separated by HPLC analysis using 75%

acetonitrile containing 0.02% trifluoroacetic acid to yield five compounds 316-HP1–5 (Fig 6). Among the five compounds, the chemical structure of 316-HP2, 3, 5 compounds that showed insecticidal activity against *P. xylostella* was determined by spectroscopic methods.

By performing ^{1}H-NMR, ^{13}C-NMR, and 2D-NMR experiments, and high-resolution electrospray ionization mass spectrometry analysis, the 316-HP2, 316-HP3, and 316-HP5 compounds were inferred as antimycin A3a, antimycin A8a, and antimycin A1a respectively (Fig 7). For antimycin A8a (316-HP3), high-resolution mass spectrometry([M+H]+, m/z 535.26) gave a molecular formula of $C_{27}H_{38}N_2O_9$. This requires that the alkyl side chain has a formula of C_5H_{11}. COSY connectivities from a methine at 2.53ppm (7-H) to methylene hydrogens at 1.65 and 1.36ppm (H_α), from Ha to methylene at 1.06ppm (H_β), from H_α to methane at 1.45ppm (H_γ), and from H_γ to a six hydrogen methyl doublet at 0.83ppm (H_α and H_β), indicated that the alkyl side chain is isopentyl (3-methyl butyl). HMBC connectivities from H_δ and H_ε to C_γ and C_β support this side-chain structure.

In the case of active substance 316-HP2 with the formula of $C_{26}H_{36}N_2O_9$ ([M+H]+, m/z 520.24), it was confirmed that it was significantly similar to the compound 316-HP3. However, there was no branched CH peak as it appeared to be a linear alkyl in the side chain, and 316-HP5 with the formula of $C_{28}H_{40}N_2O_9$ ([M+H]+, m/z 549.28) was also identified as a tautomer of 316-HP3.

3.4. Strain activity improvement through Mutagenesis–UV mutation

The number of mutant colonies decreased with the increase in exposure time. Among the tested UV mutants, the highest activity was witnessed in mutant U67. Larval mortality at 5-fold dilution broth filtrate of mutant U67 was 70% after 24 hours of treatment, and 100% after 48 hours of treatment, confirming that the insecticidal effect was higher compared to other mutants and wild type (Table 1).

In the secondary UV mutagenesis using mutant U67, the mutant U67-46 exhibited the highest larval mortality and was selected. The larvicidal activity of mutant U67-46 in the leaf disk feeding assay was 98% and 65% when treated with 20-fold and 30-fold dilutions, respectively (Table 2). In the case of mutant U67, the larval mortality was 55% when the culture filtrate was diluted 10-fold and treated, indicating that the insecticidal activity of the mutant U67-46 was increased 3 times compared to the U67, and more than 5 times compared to the wild type.

4. Discussion

To solve the unwanted effects of synthetic pesticides such as chemical pollution, accumulation of toxic materials, and development of pesticide resistance, research studies for eco-friendly compounds as alternatives have been extensively conducted and continuously utilized [25]. More specifically, insecticidal, insect growth regulatory, and antifeedant activities of actinomycetes have been studied and confirmed that actinomycetes produce a range of secondary metabolites [4, 6, 26]. In this study, larvacidal activity against *P. xylostella* was investigated in 850 actinomycetes and the larval mortality was over 80% in the initially selected 6 strains, and among them, strain KR0006 showed the highest insecticidal activity.

In the present study, three active compounds identified as antimycin A3a, antimycin A8a, and antimycin A1a were purified from the culture broth of *Streptomyces* sp. KR0006 which was identified by comparative analysis with similar bacterial strains through 16S rRNA gene sequencing. Antimycins are known as secondary metabolites produced by *Streptomyces* and other types of soil bacteria and provide competitive advantages to kill neighboring organisms such as insects, nematodes, and fungi [27]. Several studies reported that the antimycin family of compounds has exhibited a broad spectrum of insecticidal and antifungal activities [28-30]. Our results coincided with recent results by Kim, Choi (7) who had isolated five antimycins as

active compounds from *Streptomyces celluloflavus* displayed significant insecticidal activity against *P. xylostella, A. albopictus, T. urticae,* and *F. occidentalis*. In addition to insecticidal and larvicidal activity, our results revealed that the culture broth of *Streptomyces* sp. KR0006 showed a considerable oviposition suppressing activity against the Diamondback moth. Although the antifeedant, larvicidal, and growth inhibitory activity of the secondary metabolites isolated from *Streptomyces* spp. has been reported [4, 7, 30, 31], the inhibition of oviposition has not been adequately studied yet. Taking into account high-level insecticidal, larvicidal, and spawn suppressing the activities of the strain KR0006, interest has arisen in conducting additional experiments of increasing the activity of this strain.

Strain improvement can be successfully achieved through conventional selection, UV mutation, or genetic recombination [16]. The activity or productivity of several microbial strains had been enhanced by UV treatment [32-35]. It became clear from our experiments that exposure to ultraviolet (UV) irradiation (254 nm, height 17 cm) for 70 seconds to KR0006 strain improved the larvicidal activity by 70%. There are very rare studies available on UV mutation for increasing the insecticidal or herbicidal activity of metabolites produced by *Streptomyces*. However, our results were closely in agreement with research finding where two times enhancement was observed in endoglucanase activity of UV treated *Aspergillus niger* strain [36] and up to 57.4 % increase in *Streptomyces griseoaurantiacus* strain [34]. We subjected the selected mutant strain to the secondary UV irradiation which resulted in an improvement in insecticidal activity by 80 and 100 % compared with the first mutant and initial wild strains respectively. There was no back mutation observed even after the second mutation which is common in most microbe mutation studies [32]. It can be concluded that proper UV mutagenesis can be forwarded as a tool to increase the activity of secondary metabolites produced by *Streptomyces*.

In conclusion, *Streptomyces* sp. KR0006 containing antimycin A8a and its tautomers

antimycin A3a, and antimycin A1a as active substances showed high insecticidal activity against *Plutella xylostella*. The culture filtrate of the KR0006 strain showed an insecticidal and larvicidal effect by inhibiting the oviposition of moths and the growth and development of larvae. The insecticidal activity of the culture filtrate was concentration-dependent. The yield of effective insecticidal substances produced by KR0006 was increased through mutagenesis by UV radiation. The insecticidal activity of 2nd mutant U67-46 increased 3 times compared to 1st mutant U67, and more than 5 times compared to the wild type.

In our study, *Streptomyces* sp. KR0006 was found to be a valuable biological control tool against diamondback moth (*P. xylostella*) and can be used to form an environmentally safe natural product in pest management. However, further research is needed to make clear the mechanisms of insecticide action and evaluate the effectiveness in the open field.

Acknowledgments

This work was supported by Korea Institute of Planning and Evaluation for Technology in Food, Agriculture and Forestry (IPET) through Agricultural Machinery/Equipment Localization Technology Development Program, funded by Korea Institute of Planning and Evaluation for Technology in Food, Agriculture and Forestry (IPET) (321056-05).

References

1. Armes NJ, Wightman JA, Jadhav DR, Ranga Rao GV. Status of Insecticide Resistance in Spodoptera litura in Andhra Pradesh, India. Pesticide Science. 1997;50(3):240-8. doi: https://doi.org/10.1002/(SICI)1096-9063(199707)50:3<240::AID-PS579>3.0.CO;2-9.
2. Nicolopoulou-Stamati P, Maipas S, Kotampasi C, Stamatis P, Hens L. Chemical Pesticides and Human Health: The Urgent Need for a New Concept in Agriculture. Frontiers in Public Health. 2016;4. doi: 10.3389/fpubh.2016.00148.
3. Akutse K, Subramanian S, Maniania N, Dubois T, Ekesi S. Biopesticide Research and Product Development in Africa for Sustainable Agriculture and Food Security – Experiences From the International Centre of Insect Physiology and Ecology (icipe). Frontiers in Sustainable Food Systems. 2020;4. doi: 10.3389/fsufs.2020.563016.
4. Arasu MV, Al-Dhabi NA, Saritha V, Duraipandiyan V, Muthukumar C, Kim S-J. Antifeedant, larvicidal and growth inhibitory bioactivities of novel polyketide metabolite isolated from Streptomyces sp. AP-123 against Helicoverpa armigera and Spodoptera litura. BMC Microbiology. 2013;13(1):105. doi: 10.1186/1471-2180-13-105.
5. Copping LG, Menn JJ. Biopesticides: a review of their action, applications and efficacy. Pest Management Science. 2000;56(8):651-76. doi: https://doi.org/10.1002/1526-4998(200008)56:8<651::AID-PS201>3.0.CO;2-U.
6. Kaur T, Vasudev A, Sohal SK, Manhas RK. Insecticidal and growth inhibitory potential of Streptomyces hydrogenans DH16 on major pest of India, Spodoptera litura(Fab.) (Lepidoptera: Noctuidae). BMC Microbiology. 2014;14(1):227. doi: 10.1186/s12866-014-0227-1.

7. Kim JH, Choi JY, Park DH, Park D-J, Park MG, Kim SY, et al. Isolation and characterization of the insect growth regulatory substances from actinomycetes. Comparative Biochemistry and Physiology Part C: Toxicology & Pharmacology. 2020;228:108651. doi: https://doi.org/10.1016/j.cbpc.2019.108651.

8. Oka Y, Koltai H, Bar-Eyal M, Mor M, Sharon E, Chet I, et al. New strategies for the control of plant-parasitic nematodes. Pest Management Science. 2000;56(11):983-8. doi: https://doi.org/10.1002/1526-4998(200011)56:11<983::AID-PS233>3.0.CO;2-X.

9. Sharma M. Actinomycetes: Source, identification, and their applications. Int J Curr Microbiol Appl Sci. 2014;3(2):801-32.

10. Solecka J, Zajko J, Postek M, Rajnisz A. Biologically active secondary metabolites from Actinomycetes. Open Life Sciences. 2012;7(3):373-90. doi: doi:10.2478/s11535-012-0036-1.

11. Dhanasekaran D, Sakthi V, Thajuddin N, Panneerselvam A. PRELIMINARY EVALUATION OF ANOPHELES MOSQUITO LARVICIDAL EFFICACY OF MANGROVE ACTINOBACTERIA. International Journal of Applied Biology and P. 2010.

12. Montesinos E. Development, registration and commercialization of microbial pesticides for plant protection. Int Microbiol. 2003;6(4):245-52. Epub 2003/09/05. doi: 10.1007/s10123-003-0144-x. PubMed PMID: 12955583.

13. Omura S. Ivermectin: 25 years and still going strong. Int J Antimicrob Agents. 2008;31(2):91-8. Epub 2007/11/27. doi: 10.1016/j.ijantimicag.2007.08.023. PubMed PMID: 18037274.

14. Osman GMS, Sonya HM. ANTAGONISTIC AND INSECTICIDAL ACTIVITIES OF SOME STREPTOMYCES ISOLATES. Pakistan Journal of Biotechnology. 2021;4((1-2)):65-71.

15. Miller JH. Mutational specificity in bacteria. Annu Rev Genet. 1983;17:215-38. Epub 1983/01/01. doi: 10.1146/annurev.ge.17.120183.001243. PubMed PMID: 6364961.
16. Parekh S, Vinci VA, Strobel RJ. Improvement of microbial strains and fermentation processes. Appl Microbiol Biotechnol. 2000;54(3):287-301. Epub 2000/10/13. doi: 10.1007/s002530000403. PubMed PMID: 11030563.
17. Siddique S, Syed Q, Adnan A, Qureshi FA. Production and Screening of High Yield Avermectin B1b Mutant of Streptomyces avermitilis 41445 Through Mutagenesis. Jundishapur J Microbiol. 2014;7(2):e8626-e. Epub 2014/02/01. doi: 10.5812/jjm.8626. PubMed PMID: 25147669.
18. OriginLab. Origin(Pro), 81. Northampton, Massachusetts, USA.: OriginLab corporation Northampton, Massachusetts, USA.; 2021. p. www.OriginLab.com.
19. Bo AB, Kim JD, Kim YS, Sin HT, Kim HJ, Khaitov B, et al. Isolation, identification and characterization of Streptomyces metabolites as a potential bioherbicide. PLoS One. 2019;14(9):e0222933. Epub 2019/09/24. doi: 10.1371/journal.pone.0222933. PubMed PMID: 31545849; PubMed Central PMCID: PMCPMC6756554.
20. Saitou N, Nei M. The neighbor-joining method: a new method for reconstructing phylogenetic trees. Molecular Biology and Evolution. 1987;4(4):406-25. doi: 10.1093/oxfordjournals.molbev.a040454.
21. Tamura K, Stecher G, Peterson D, Filipski A, Kumar S. MEGA6: Molecular Evolutionary Genetics Analysis Version 6.0. Molecular Biology and Evolution. 2013;30(12):2725-9. doi: 10.1093/molbev/mst197.
22. Kim HJ, Bo AB, Kim JD, Kim YS, Khaitov B, Ko Y-K, et al. Herbicidal Characteristics and Structural Identification of the Potential Active Compounds from Streptomyces sp. KRA17-580. Journal of Agricultural and Food Chemistry. 2020;68(52):15373-80. doi: 10.1021/acs.jafc.0c01974.

23. Khattab AA, & Mohamed, S. A. A. . Mutation induction and protoplast fusion of Streptomyces spp. for enhanced alkaline protease production. . Journal of Applied Sciences Research. 2012;8(2):807-14. doi: https://doi:10.3923/biotech.2008.456.462.

24. Wang LY, Huang ZL, Li G, Zhao HX, Xing XH, Sun WT, et al. Novel mutation breeding method for Streptomyces avermitilis using an atmospheric pressure glow discharge plasma. J Appl Microbiol. 2010;108(3):851-8. Epub 20090720. doi: 10.1111/j.1365-2672.2009.04483.x. PubMed PMID: 19735332.

25. Cantrell CL, Dayan FE, Duke SO. Natural Products As Sources for New Pesticides. Journal of Natural Products. 2012;75(6):1231-42. doi: 10.1021/np300024u.

26. Xu L, Liang K, Duan B, Yu M, Meng W, Wang Q, et al. A Novel Insecticidal Peptide SLP1 Produced by Streptomyces laindensis H008 against Lipaphis erysimi. Molecules. 2016;21(8):1101. PubMed PMID: doi:10.3390/molecules21081101.

27. Seipke RF, Hutchings MI. The regulation and biosynthesis of antimycins. Beilstein Journal of Organic Chemistry. 2013;9:2556-63. doi: 10.3762/bjoc.9.290.

28. Hosotani N, Kumagai K, Nakagawa H, Shimatani T, Saji I. Antimycins A10 approximately A16, seven new antimycin antibiotics produced by Streptomyces spp. SPA-10191 and SPA-8893. J Antibiot (Tokyo). 2005;58(7):460-7. Epub 2005/09/16. doi: 10.1038/ja.2005.61. PubMed PMID: 16161485.

29. Shiomi K, Hatae K, Hatano H, Matsumoto A, Takahashi Y, Jiang C-L, et al. A New Antibiotic, Antimycin A9, Produced by Streptomyces sp. K01-0031. The Journal of Antibiotics. 2005;58(1):74-8. doi: 10.1038/ja.2005.10.

30. Yan L-L, Han N-N, Zhang Y-Q, Yu L-Y, Chen J, Wei Y-Z, et al. Antimycin A18 produced by an endophytic Streptomyces albidoflavus isolated from a mangrove plant. The Journal of Antibiotics. 2010;63(5):259-61. doi: 10.1038/ja.2010.21.

31. Samri SE, Baz M, Ghalbane I, El Messoussi S, Zitouni A, El Meziane A, et al. Insecticidal activity of a Moroccan strain of Streptomyces phaeochromogenes LD-37 on larvae, pupae and adults of the Mediterranean fruit fly, Ceratitis capitata (Diptera: Tephritidae). Bulletin of Entomological Research. 2017;107(2):217-24. doi: 10.1017/S000748531600078X.

32. Chand P, Aruna A, Maqsood AM, Rao LV. Novel mutation method for increased cellulase production. Journal of Applied Microbiology. 2005;98(2):318-23. doi: https://doi.org/10.1111/j.1365-2672.2004.02453.x.

33. Ikehata H, Ono T. The Mechanisms of UV Mutagenesis. Journal of Radiation Research. 2011;52(2):115-25. doi: 10.1269/jrr.10175.

34. Kumar AK. UV mutagenesis treatment for improved production of endoglucanase and β-glucosidase from newly isolated thermotolerant actinomycetes, Streptomyces griseoaurantiacus. Bioresources and Bioprocessing. 2015;2(1):22. doi: 10.1186/s40643-015-0052-x.

35. Yao R, Li M, Deng S, Hu H, Wang H, Li F. Mutagenesis ofTrichoderma Virideby Ultraviolet and Plasma. Plasma Science and Technology. 2012;14(4):353-6. doi: 10.1088/1009-0630/14/4/16.

36. Irfan M, Syed Q, Javed J. UV mutagenesis of aspergillus niger for enzyme production in submerged fermentation. Pakistan Journal of Biochemistry and Molecular Biology. 2011;44(4):137-40.

406

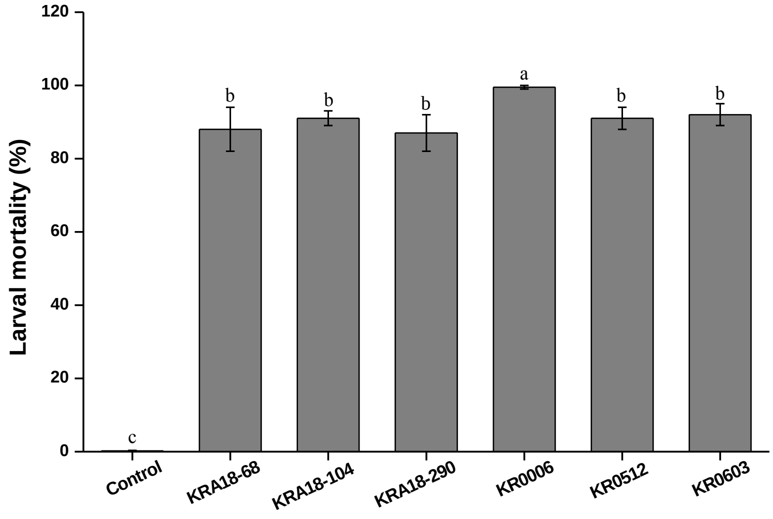

Fig. 1. Insecticidal activity of the actinobacteria strains against *Plutella xylostella*. Second instar larvae of *P. xylostella* were treated with undiluted culture filtrate of each actinobacterium strain using the cabbage leaf dipping method and the mortality was evaluated at 3 days after treatment. Different letters above the error bars indicate a significant difference in the Fisher's LSD test ($P < 0.05$).

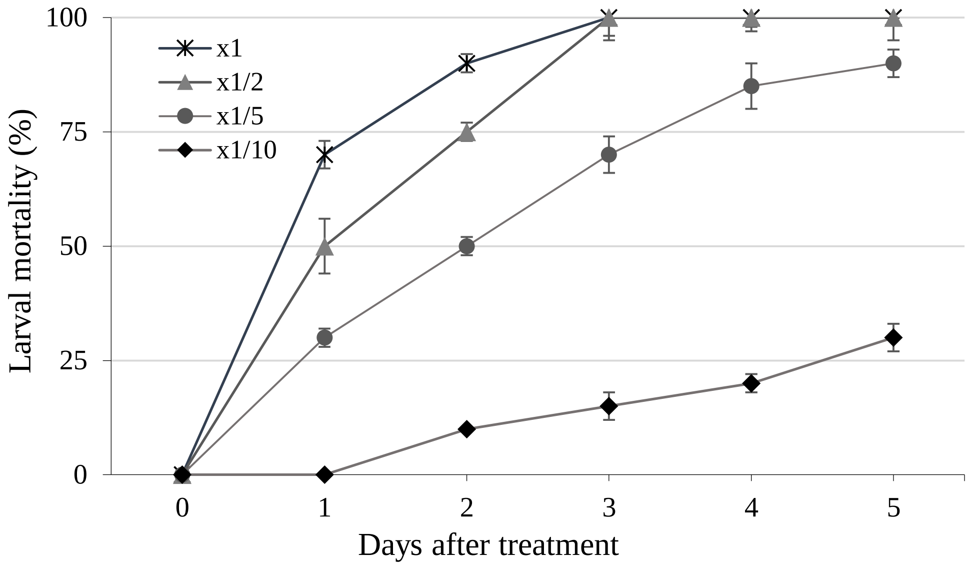

Fig. 2. Concentration-dependent insecticidal activities of KR0006 against *Plutella xylostella*. The 10mL diluted solutions of the KR0006 were sprayed on the foliage of Chinese cabbage (2 plants) growing in the greenhouse. Plants were inoculated with *P. xylostella* 2nd instars (10 larvae/plant) and the mortality was calculated 5 days after treatment. The vertical bars indicate the standard error of the mean.

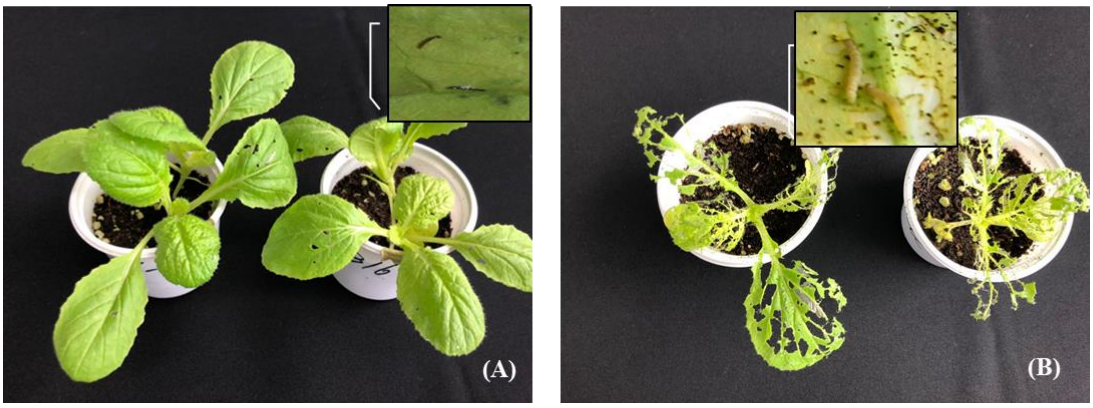

Fig. 3. Insecticidal activities of the broth filtrate of KR0006 against *Plutella xylostella* 2nd instars in greenhouse conditions. Representative pictures were taken 5 days after treatment (DAT). (A); 2-fold dilution of broth filtrate, (B); untreated control.

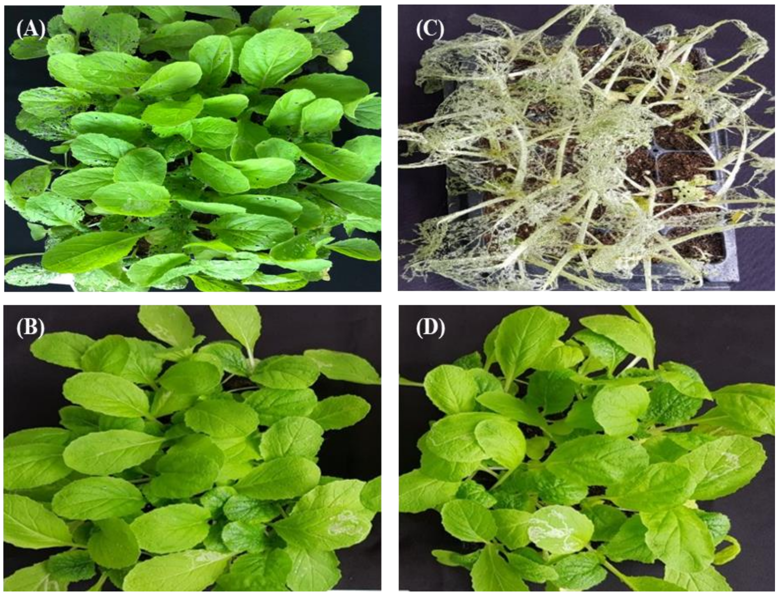

Fig. 4. The ovipositional repellency and larvicidal activity of *Streptomyces* sp. KR0006 against diamondback moth. The culture filtrate was first sprayed on the cabbage seedlings grown in the tray, and then 50 diamondback moths were inoculated. 6 days after treatment (DAT), the culture filtrate was secondarily applied to half of the tray. (A); 6 DAT control, (B); 1st treatment of broth filtrate, (C); 10 DAT control, (D); 2nd treatment of broth filtrate.

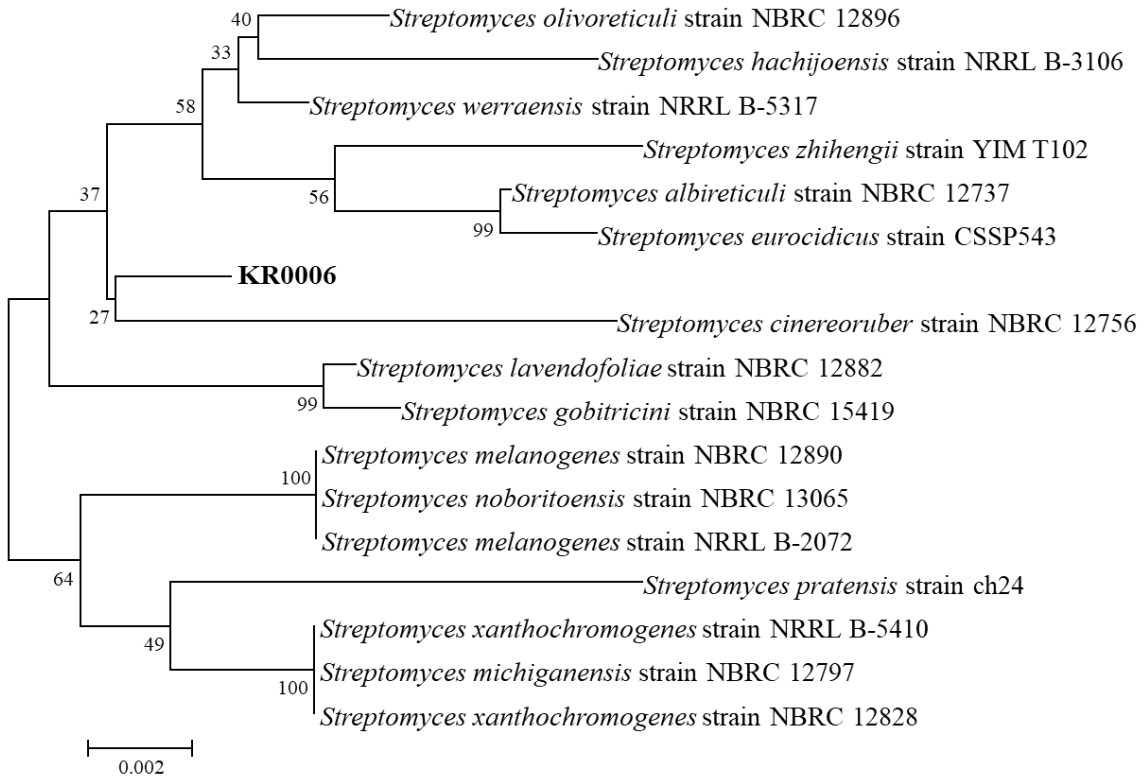

Fig. 5. Neighbor-joining tree of strain KR0006 based on its 16S rRNA gene sequence showing the phylogenetic relationship with related *Streptomyces* species. Numbers at each branch node indicate the bootstrap percentage of 1000 replications.

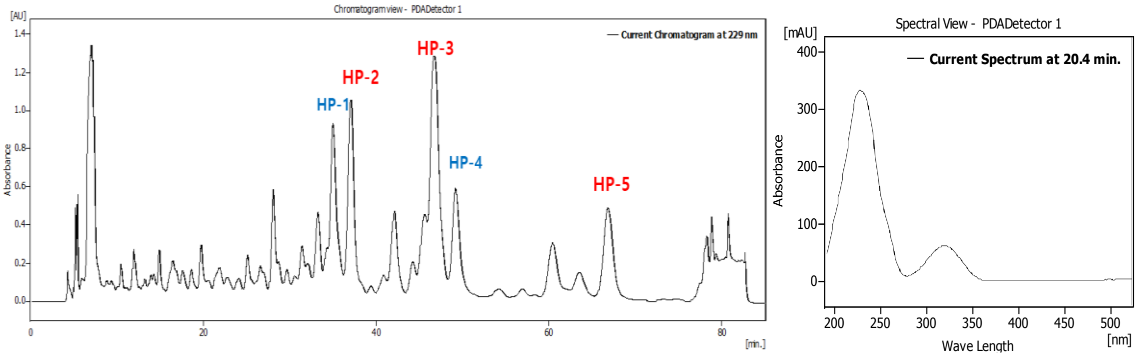

Fig. 6. High-performance liquid chromatography chromatogram of compounds 1~5 produced by *Streptomyces* sp. KR0006 and UV spectrum. Column, waters Atlantis T3 column (i.d. 4.6x250 mm); mobile phase, 70% acetonitrile/0.02% TFA; flow rate, 0.8 ml/min; detection, 230nm.

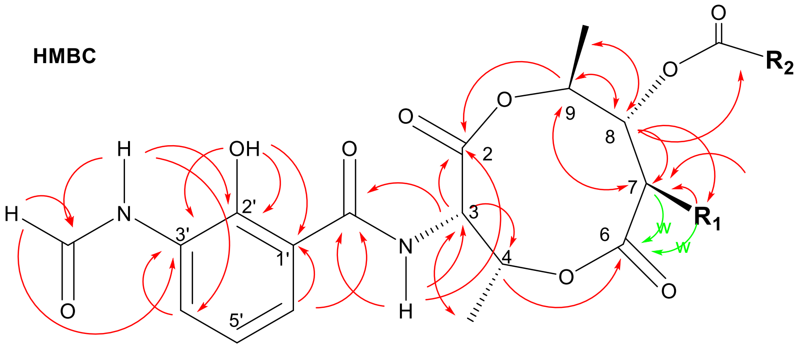

	MW	R1	R2
Antimycin A3a (316-HP2)	519	$(CH_2)_3CH_3$	$CH(CH_3)CH_2CH_3$
Antimycin A8a (316-HP3)	534	$(CH_2)_2CH(CH_3)_2$	$CH(CH_3)CH_2CH_3$
Antimycin A1a (316-HP5)	548	$(CH_2)_5CH_3$	$CH(CH_3)CH_2CH_3$

Fig. 7. The structures of the antimycins purified from *Streptomyces* sp. KR0006.

Table. 1. Insecticidal activity of the mutants against *Plutella xylostella* larvae. To estimate the larvicidal activity, Chinese cabbage leaf disks (diameter 3 cm) were dipped for 30 seconds in 5-fold diluted (x1/5) each mutant and wild broth filtrate solutions. Once the leaf disks dried they were inoculated with *P. xylostella* 2nd instars (10 larvae/disk). Values are the mean of three replication and different letters in each column indicate significant differences in the Fisher's LSD test (P < 0.05).

Treatments	Larval mortality (%)		
	After 24h	48h	72h
Mutant - U21	0±0e	60±5c	95±4a
U22	0±0e	70±5b	70±5b
U23	30±2c	75±5b	100±0a
U24	0±0e	50±4c	80±10b
U25	0±0e	15±3d	92±2a
U26	0±0e	0±0e	100±0a
….	...	…	…
U67	70±5a	100±0a	100±0a
U73	50±5b	80±10b	100±0a
….	...	…	….
Wild type	5±2d	20±5d	45±5c

Table. 2. Insecticidal activity of KR0006 (WT) and mutants (first and second) against *Plutella xylostella*. Values are the mean of three replication and different letters in each column indicate significant differences in the Fisher's LSD test (P < 0.05).

Dilution fold	Larval mortality (%)		
	KR0006 (WT)	U67	U67-46
1	100±0a	100±0a	100±0a
2	100±0a	100±0a	100±0a
5	90±3a	100±0a	100±0a
10	15±4b	55±3b	100±0a
20	0±0c	25±3c	98±2a
30	0±0c	0±0d	65±10b
