# Supplementary material for: Insecticidal characteristics and structural identification of the potential active compounds from Streptomyces sp. KR0006: Strain improvement through mutagenesis
Source: PLoS One. 2022 Sep 26;17(9):e0274766. doi: 10.1371/journal.pone.0274766 (PMC9512179; doi:10.1371/journal.pone.0274766)
Supplement: Attachment — Submitted filename: Response to Reviewers.docx [file pone.0274766.s003.docx]

**Journal Requirements:**

When submitting your revision, we need you to address these additional requirements.

1. Please ensure that your manuscript meets PLOS ONE's style requirements, including those for file naming. The PLOS ONE style templates can be found at
   <https://journals.plos.org/plosone/s/file?id=wjVg/PLOSOne_formatting_sample_main_body.pdf> and 
   <https://journals.plos.org/plosone/s/file?id=ba62/PLOSOne_formatting_sample_title_authors_affiliations.pdf>

- **After carefully reading the formatting style, the authors followed wholly to meet the manuscript PLOS ONE’s style requirements.**

2. Financial disclosure:

- **This work was supported by Korea Institute of Planning and Evaluation for Technology in Food, Agriculture and Forestry (IPET) through Agricultural Machinery/Equipment Localization Technology Development Program, funded by Korea Institute of Planning and Evaluation for Technology in Food, Agriculture and Forestry (IPET) (321056-05). However, the funder had no role in study design, data collection, and analysis, decision to publish, or preparation of the manuscript. This manuscript should be published in OPEN ACCESS. I will state the role of the Funder statement in the cover letter too and please change the online submission form on our behalf.**

**Additional Editor Comments:**

3. The manuscript needs substation revision and the reference section should be updated and discuss more bio-pesticides including microbial pesticides in the introduction as well in the discussion least the author should refer to the following documents and rewrite the advantages of biocontrol potential of bio-pesticides. Please refer to several articles published recently in Plos One.
   I suggest the author go through all the points suggested by the reviewer and provide a point-by-point explanation about your revision. In not please provide a suitable rebuttal to avoid the next round of revision.
   Introduction:
   Please refer my first point, it needs more information about biopesticides through microbial culture with recent updates. # refer doi:10.3390/jof6040196, 10.1016/j.jip.2018.10.008, 10.1016/j.ecoenv.2019.109474, 0.1007/978-81-322-2056-5_3,
   The result, discussion, and Conclusion section need to develop according to your result and appropriate citations must be provided to justify your claim.

- **Thank you for your valuable comments. The manuscript was revised and references and all the citations were also updated. In the introduction and discussion sections, the authors tried to discuss more detail bio-pesticides, especially microbial pesticides. Authors read all the literature suggested by the editor and reviewers and cited where needed. A number of articles published in PLOS ONE were also cited in the manuscript. Almost all the sections of the manuscript were rewritten and the manuscript was developed (refer to the manuscript file).**

**Review’s Comments to the Author**

Reviewer #2: There are several aspects that need clarifications/implementation.
Please refer to the observations/suggestions available as notes in the enclosed file.

The authors should also check the following references since are related to the same topic:

Cao, Haijing, et al. "5′-Epi-SPA-6952A, a new insecticidal 24-membered macrolide produced by *Streptomyces diastatochromogenes* SSPRC-11339." Natural product research 33.5 (2019): 659-664.

Gopalakrishnan, Subramaniam, et al. "Insecticidal activity of a novel fatty acid amide derivative from *Streptomyces* species against *Helicoverpa armigera*." Natural Product Research 30.24 (2016): 2760-2769.

Tanvir, Rabia, Imran Sajid, and Shahida Hasnain. "Larvicidal potential of Asteraceae family endophytic actinomycetes against *Culex quinquefasciatus* mosquito larvae." Natural Product Research 28.22 (2014): 2048-2052.

- **Thank you for your valuable comments. The authors read carefully the suggested papers by the reviewers and cited them where needed. The authors took into consideration the suggestions and comments in the enclosed file given by the reviewers and the manuscript was revised according to those.**
