# Supplementary material for: Insecticidal characteristics and structural identification of the potential active compounds from Streptomyces sp. KR0006: Strain improvement through mutagenesis
Source: PLoS One. 2022 Sep 26;17(9):e0274766. doi: 10.1371/journal.pone.0274766 (PMC9512179; doi:10.1371/journal.pone.0274766)
Supplement: Attachment — Submitted filename: Response to Reviewers.docx [file pone.0274766.s005.docx]

**Journal Requirements:**

When submitting your revision, we need you to address these additional requirements.

1. Please ensure that your manuscript meets PLOS ONE's style requirements, including those for file naming. The PLOS ONE style templates can be found at
   <https://journals.plos.org/plosone/s/file?id=wjVg/PLOSOne_formatting_sample_main_body.pdf> and 
   <https://journals.plos.org/plosone/s/file?id=ba62/PLOSOne_formatting_sample_title_authors_affiliations.pdf>

- **After carefully reading the formatting style, the authors followed wholly to meet the manuscript PLOS ONE’s style requirements.**

2. Financial disclosure:

- **The funders had no role in study design, data collection, and analysis, decision to publish, or preparation of the manuscript.**

3. The guidelines for resubmitting your figure files

- **Thank you for showing us the guideline for adjustments of figure files. Now, all figure files are adjusted to the journal requirements and resubmitted.**

**Review’s Comments to the Author**

1 80- it should be better to report the G value instead rpm
2 115- did you compare the results also with those available on Genebank?
3 205- you should compare the experimental data with those reported in literature for these compounds since are all already described. The relevant references should be provided.
4 209- if you are referring to the molecular formula it should be considered that the species [M+H]+ is the protonated form of and its empirical formula is different. It is formally one ion positively charged. It should be more clear.
5 Fig7- HMBC correlations cannot be checked without the experimental spectra (mono and bidimensional NMR)

- **Thank you for your valuable comments.**

1 **Rpm value of centrifuge was changed into g value in other word RCF (Relative Centrifugal Force)**
2 **Yes, obtained sequences of the secured 16S rRNA gene were analyzed through the BLASTn program of NCBI (**[**http://www.ncbi.nlm.nih.gov**](http://www.ncbi.nlm.nih.gov)**).**
3 **Yes, we compared the experimental data with those reported in the literature for these compounds, and relevant references were cited where they are needed. Please refer to the manuscript file.**
4 **Thank you for your valuable comment. The structural determination part of the manuscript was deeply reviewed and revised. Please refer to the tracked manuscript or manuscript file to see corrections.**
5 **Figure seven is now provided with the experimental spectra (mono and bidimensional NMR).**
